# The Effect of Lactoferrin and Magnesium Phosphate Hydrogel Coating on Osteoblast Activity on Titanium Discs: An In Vitro Study

**DOI:** 10.7759/cureus.115441

**Published:** 2026-08-30

**Authors:** Kritika Patni, Srilakshmi J, Krishna Kumar U

**Affiliations:** 1 Department of Prosthodontics, Rajarajeswari Dental College and Hospital, Bengaluru, IND

**Keywords:** hydrogels, lactoferrin, magnesium phosphate, osseointegration, osteoblasts

## Abstract

Background and objective

The clinical success of modern implantology is largely based on the concept of osseointegration. The primary challenge is to develop multifunctional surfaces that can simultaneously promote rapid bone formation while ensuring safety and biocompatibility. This study aimed to demonstrate the biocompatibility and cytotoxicity profiles of a hydrogel composed of lactoferrin (LF) and magnesium phosphate and to assess its safety for clinical use in promoting osseointegration. The key objective was to evaluate the osteoblastic activity and cell proliferation induced by this surface modification.

Materials and methods

In this study, 16 commercially pure grade 4 titanium discs (5 mm in diameter, 2 mm in thickness) were divided into two groups, comprising eight samples each. The experimental group comprised titanium discs coated by a dip-coating technique with an LF- and magnesium phosphate-loaded hydrogel, while the second group served as an uncoated control. Biocompatibility and cell viability were evaluated using the MTT assay in the osteosarcoma-derived MG-63 cell line. Alkaline phosphatase (ALP) activity was measured to assess early osteoblastic differentiation and the mineralization potential of the hydrogel-coated Ti discs compared to those of the control group.

Results

The LF- and magnesium phosphate hydrogel-coated titanium discs exhibited significantly higher metabolic activity and cell viability compared to the uncoated control group at all time points (p < 0.001). By Day seven, the LF-magnesium phosphate hydrogel combination coating demonstrated significantly higher cellular metabolic activity, reaching a peak optical density (OD) of 1.173 ± 0.030 at 570 nm, compared to 0.891 ± 0.025 for the uncoated control (p < 0.001). Morphological analysis showed a 1.76-fold increase in cell spreading area within 24 hours, indicating improved initial adhesion. Furthermore, ALP activity was markedly elevated in the experimental group, with values reaching 819 ± 28 U/L by Day seven, indicating enhanced early osteoblastic differentiation.

Conclusions

There was a significantly enhanced biological response of osteosarcoma-derived MG-63 cells on titanium substrates with the hydrogel coating. The modified discs outperformed the control in terms of cytocompatibility, cell proliferation, and osteogenic functional activity. These findings suggest that the integration of an LF- and magnesium phosphate-loaded hydrogel can enhance initial cell attachment and promote faster osseointegration, offering a promising approach for dental implant surface modification. While these results are promising for dental implant technology, further research is needed to fully validate its clinical efficacy.

## Introduction

The clinical success of modern implantology is rooted in the concept of osseointegration, a term first coined by Professor Per-Ingvar Brånemark in the 1950s [1]. This discovery was famously serendipitous; while studying blood circulation in rabbit femurs using titanium chambers, Brånemark observed that the metal had become so firmly affixed to the living bone that it could not be removed [2]. He defined osseointegration as a direct structural and functional connection between ordered, living bone and the surface of a load-carrying implant [2]. This breakthrough shifted dental practice from mechanical retention methods to a biological paradigm in which the implant surface becomes an integrated part of the host’s skeletal system [3]. Since the first human clinical application in 1965, millions of patients have benefited from this approach, though the challenge remains to accelerate this process in patients with compromised bone quality [1].

Titanium and its alloys have remained the "gold standard" for dental and orthopedic implants for decades due to their exceptional biocompatibility, corrosion resistance, and mechanical strength [4]. Among the various types, commercially pure grade 4 titanium is the most frequently used in dental applications because it offers the highest static strength and moderate plasticity compared to lower grades [5]. Despite these advantages, titanium is inherently bioinert. Its surface is naturally covered by a thin titanium oxide layer that, while stable, does not actively promote the migration, adhesion, or differentiation of osteoblast progenitors [6]. This lack of bioactivity can prolong healing times and lead to early-stage failures, necessitating surface modifications that transform the bioinert interface into a bioactive one [7].

Lactoferrin (LF) is an 80 kDa iron-binding glycoprotein that has recently emerged as a potent therapeutic agent for bone regeneration [8]. Naturally found in mucosal secretions such as milk and saliva, LF exhibits a broad spectrum of activities, including antimicrobial, anti-inflammatory, and immunomodulatory effects [8]. In bone metabolism, LF acts as a potent mitogen; it has been shown to activate the extracellular signal-regulated kinase pathway in osteoblasts through the low-density lipoprotein receptor-related protein [9]. By stimulating the proliferation and differentiation of primary osteoblasts and increasing the expression of bone morphogenetic proteins, LF provides an anabolic stimulus that significantly enhances the biological response at implant surfaces [8].

Magnesium phosphate is a biocompatible mineral phase that closely mimics the density and mineral composition of human cortical bone [10]. Magnesium itself is an essential element for bone health, as its presence positively influences bone tissue growth and activates bone cells [11]. Incorporating magnesium into titanium coatings has been shown to accelerate apatite formation and significantly improve initial protein adsorption and cell spreading [11]. Unlike traditional bioinert materials, magnesium-based coatings release Mg ions that can modulate the local pH and ionic environment, thereby creating a favorable niche for early cellular attachment and subsequent mineralization [12].

The primary challenge in contemporary implantology is to develop multifunctional surfaces that can simultaneously promote rapid bone formation while ensuring safety and biocompatibility [13]. While the individual osteogenic and bone-regenerative capacities of LF and magnesium phosphate hydrogels are well-documented in the existing literature, the biological potential of their combined application on titanium substrates remains to be fully explored. This study aims to evaluate the biological response of human osteosarcoma-derived MG-63 osteoblast-like cells to titanium discs coated with a specialized hydrogel loaded with both LF and magnesium phosphate. By assessing parameters such as cell viability through MTT assays, morphological spreading, and alkaline phosphatase (ALP) activity, we seek to determine whether this dual-agent modification can significantly enhance initial osseointegration compared to conventional uncoated titanium.

## Materials and methods

Study design and group allocation

Commercially pure grade 4 titanium discs measuring 5 mm in diameter and 2 mm in thickness were selected for the study. A total of 16 samples were utilized. The experimental design comprised two distinct cohorts evaluated within a single, controlled cell culture run, where the discs served as technical replicates (n = 8 per group): an active treatment group receiving LF-magnesium phosphate hydrogel-coated titanium discs, and a control group receiving untreated titanium discs. Cytotoxicity and viability of human osteosarcoma-derived MG-63 osteoblast-like cells were assessed. The MTT assay was performed to evaluate cell viability, while the ALP assay was conducted strictly to evaluate early osteogenic differentiation and osteoblast functional activity.

Lactoferrin-magnesium phosphate hydrogel formulation

A LF hydrogel with a concentration of 100 µg/ml, Mg phosphate ion concentration of 10 mM, and gelatin as a stabilizer was formulated. LF powder was dissolved in phosphate-buffered saline (PBS) to achieve a final concentration of 100 µg/ml. The resulting solution was stirred continuously until complete dissolution of the protein was achieved. Gelatin (0.5%-2% w/v) was utilized as a stabilizing agent. The gelatin solution was prepared by dissolving the required mass of powder in PBS under gentle agitation to prevent the formation of air bubbles while ensuring complete dissolution.

Magnesium phosphate salts were dissolved in PBS to achieve a final concentration of 10 mM Mg²⁺. The solution was stirred continuously until complete dissolution was observed. The hydrogel-forming polymer solution was prepared by dissolving the polymer powder in PBS and stirring until a uniform precursor solution was obtained. The previously prepared LF solution was incorporated into the hydrogel precursor under gentle agitation. Continuous stirring was maintained to ensure the homogeneous distribution of LF throughout the polymer matrix. Subsequently, the gelatin and magnesium phosphate solutions were added to the LF-hydrogel mixture. Following the incorporation of all components, the precursor solution transitioned into a solid hydrogel via cooling, ensuring the formation of a stable and uniform matrix.

Surface preparation and dip-coating technique

To ensure the removal of any surface contaminants or manufacturing residues, the titanium discs were subjected to a sequential ultrasonic cleaning protocol. Each disc was immersed in high-purity acetone, followed by absolute ethanol, and finally in sterile deionized water. The discs were processed in an ultrasonic cleaner for 10 minutes per solvent cycle. Following sterilization, the discs were aseptically transferred to a sterile 24-well culture plate. The plates were then sealed and maintained under sterile conditions to prevent any microbial contamination prior to the coating and seeding phases. The coating process was performed using a dip-coating technique, where discs were immersed in the LF-magnesium phosphate hydrogel solution and subsequently air-dried for 10 minutes.

MTT assay for cell viability

To ensure consistency across all experimental groups, human osteosarcoma-derived MG-63 osteoblast-like cells were harvested and resuspended at a specific concentration. Each titanium disc, previously prepared in a sterile 4-well culture plate, was seeded with a density of 2.0 × 10⁴ cells per disc. This density was selected to allow for optimal cell-to-surface interaction and subsequent proliferation.

The cell-seeded discs were maintained in a controlled incubator environment at a constant temperature of 37°C. The atmospheric conditions were strictly regulated with a 5% CO₂ concentration to maintain physiological pH levels. The growth medium utilized was Dulbecco's modified Eagle's medium (DMEM), supplemented with 10% fetal bovine serum (FBS) for nutritional support and 1% penicillin to prevent microbial contamination. To provide a continuous supply of nutrients and remove metabolic waste products, a systematic media exchange was performed. The culture medium was aspirated and replaced with fresh, pre-warmed DMEM every 48 hours throughout the duration of the incubation period.

The cells were incubated in the presence of the LF-magnesium phosphate hydrogel-coated discs (experimental group) and untreated titanium discs (control group) for durations of 24, 72, and 168 hours. At each designated time point, the culture medium was removed and replaced with a fresh medium containing the MTT reagent to achieve a final concentration of 0.5 mg/ml. The plates were incubated for four hours at 37°C, allowing the mitochondrial dehydrogenases of viable cells to reduce the yellow tetrazolium salt into insoluble purple formazan crystals.

Post-incubation, the supernatant was carefully aspirated, and 100 µl of Dimethyl Sulfoxide (DMSO) was added to each well to solubilize the crystals. The plates were placed on an orbital shaker for 10 minutes to ensure complete homogenization. The metabolic activity was quantified by measuring the optical density (OD) of the solution using a microplate reader at a wavelength of 570 nm. The intensity of the colored product was directly proportional to the number of viable cells present on the titanium discs.

Relative cell viability was calculated using the formula:

Relative viability (%) = $\displaystyle{\displaylines{(OD sample/ OD control Day 1) X 100}}$

Estimated cell counts were derived from a standard curve using the equation:

Estimated cells/disc = $\displaystyle{\displaylines{(OD/0.35) X 20,000}}$

Alkaline phosphatase (ALP) activity assay

To evaluate the early osteogenic differentiation of the MG-63 cells, ALP activity was quantified at critical time points (Day one and Day seven). Following incubation, the titanium discs were rinsed with sterile PBS, and the adherent cells were subjected to a standardized lysis protocol to release the intracellular enzymes into a stabilized lysate for biochemical analysis.

A series of p-Nitrophenyl phosphate (pNPP) standards were prepared in a 96-well plate with a final volume of 120 µL per well. MG-63 cell lysates or supernatants were added to the wells at volumes ranging from 2 to 80 µL, with the total volume adjusted to 80 µL using assay buffer. To eliminate endogenous ALP interference in the background controls, 20 µL of Stop Solution was added to these specific wells prior to substrate addition. For substrate incorporation, 50 µL of 5 mM pNPP solution was added to all sample and background control wells. To initiate the reaction in the standard wells, 10 µL of ALP enzyme solution was added to each.

The plate was incubated at 25°C for 60 minutes in a dark environment to prevent light-induced degradation. During this period, the ALP enzyme catalyzed the hydrolysis of the colorless pNPP substrate into colored p-Nitrophenol (pNP). The enzymatic reaction was halted in all remaining sample and standard wells by the addition of 20 µL of Stop Solution. After brief agitation to ensure homogeneity, the absorbance (OD) was measured at 405 nm using a microplate reader. The net ALP activity was determined by subtracting the absorbance of the sample background control from the sample wells. Activity was then quantified by comparing the corrected absorbance values against the pNP standard curve.

Sample size estimation

Sample size estimation was performed prior to the study using G*Power software (version 3.1.9.7; Heinrich-Heine-Universität Düsseldorf, Düsseldorf, Germany). Guided by preliminary screening data and established literature on biofunctionalized titanium coatings, a large effect size (Cohen’s d = 1.60) was anticipated for initial cellular responses between functionalized and uncoated titanium surfaces. Assuming a two-tailed alpha error of 5% (alpha = 0.05) and a statistical power of 80% (1-beta = 0.80), the analysis revealed that a minimum of 16 samples would be necessary for the present study. Thus, each study group comprised n = 8 technical replicates.

Statistical analysis

SPSS Statistics for Windows Version 22.0 (IBM Corp., Armonk, NY) was used to perform all statistical analyses. Descriptive analysis of all explanatory and outcome parameters was conducted using frequencies and proportions for categorical variables, and mean ± standard deviation (SD) for continuous variables. Independent Student's t-tests were applied to compare mean absorbance values for the MTT assay between the test and control groups at different time intervals. This test was also used to compare mean ALP activity and mean cell area per disc between the two groups at corresponding intervals. Paired sample t-tests were utilized to evaluate changes in mean ALP activity and mean cell area per disc within each group between earlier and later intervals. Repeated Measures ANOVA followed by Bonferroni’s post-hoc test was employed to assess changes in mean absorbance values for the MTT assay within each group across multiple time intervals. The level of statistical significance was set at p < 0.05.

## Results

As shown in Table 1, on Day one, the mean absorbance value (OD at 570 nm) for the LF and magnesium phosphate hydrogel combination coating group was 0.435 ± 0.016, while the uncoated control group showed a mean of 0.359 ± 0.012. The difference of 0.076 was statistically significant (p < 0.001), indicating that MG-63 cell viability was already higher on the coated discs at the earliest interval. By Day three, the combination coating group demonstrated a mean absorbance of 0.764 ± 0.027, whereas the control group recorded 0.589 ± 0.020. The mean difference of 0.175 was again statistically significant (p < 0.001). This showed that cell proliferation continued to increase more prominently on the modified discs. On Day seven, the combination coating group reached a peak mean absorbance of 1.173 ± 0.029, while the control group reached 0.891 ± 0.024 (p < 0.001). When expressed as relative cell viability, the combination coating group reached 326.8% ± 8.4% by Day seven, compared to 248.3% ± 7.0% for the uncoated control. This confirmed that the LF and magnesium phosphate hydrogel coating consistently supported greater cell viability and proliferation across the study period, with the effect becoming more pronounced at later intervals.

**Table 1:** Comparison of mean absorbance values (OD at 570 nm) for MTT assay between two groups at different time intervals using independent Student's t-test *Statistically significant Mean difference = mean value at test - mean value at control OD: optical density; SD: standard deviation

Time	Groups	N	Mean	SD	Mean Diff.	P-value	t-statistic value
Day 1	Test	8	0.435	0.016	0.076	< 0.001^*^	10.748
	Control	8	0.359	0.012			
Day 3	Test	8	0.764	0.027	0.175	< 0.001^*^	14.731
	Control	8	0.589	0.02			
Day 7	Test	8	1.173	0.029	0.281	< 0.001^*^	21.189
	Control	8	0.891	0.024			

As shown in Table 2, on Day one, the mean ALP activity in the LF and magnesium phosphate hydrogel combination coating group was 497.50 ± 22.52 U/L, while the control group showed a mean of 335.00 ± 16.04 U/L. The mean difference of 162.50 U/L was statistically significant (p < 0.001). This indicated that early osteogenic differentiation and osteoblast functional activity were considerably higher on the coated discs compared to the uncoated controls at the earliest interval. By Day seven, the combination coating group demonstrated a mean ALP activity of 818.75 ± 29.00 U/L, whereas the control group recorded 586.25 ± 16.85 U/L. The mean difference of 232.50 U/L was statistically significant (p < 0.001). This demonstrated that early osteogenic differentiation continued to increase more prominently on the coated discs, reflecting sustained functional maturation over time. Overall, the modified titanium discs consistently showed significantly higher ALP activity at both time points.

**Table 2:** Comparison of mean ALP activity (U/L) between two groups at different time intervals using independent Student's t-test ^*^Statistically significant Mean difference = mean value at test - mean value at control ALP: alkaline phosphatase; SD: standard deviation

Time	Groups	N	Mean	SD	Mean Diff.	p-value	t-statistic value
Day 1	Test	8	497.5	22.52	162.5	< 0.001^*^	16.625
	Control	8	335	16.036			
Day 7	Test	8	818.75	29.001	232.5	< 0.001^*^	19.606
	Control	8	586.25	16.85			

As detailed in Table 3, at 24 hours, the mean cell area per disc in the LF and magnesium phosphate hydrogel combination coating group was 2145.00 ± 125.81 μm², while the control group showed a mean of 1221.25 ± 67.07 μm². The mean difference of 923.75 μm² was statistically significant (p < 0.001). This indicated that MG-63 cells spread more extensively on the coated discs at the earliest interval, suggesting that the dual hydrogel coating promoted superior early cell adhesion. At 72 hours, the combination coating group demonstrated a mean cell area of 3756.25 ± 177.28 μm², whereas the control group recorded 2340.00 ± 128.73 μm². The mean difference of 1416.25 μm² was statistically significant (p < 0.001). This confirmed that the LF and magnesium phosphate combination coating enhanced cell adhesion and spreading in a time-dependent manner.

**Table 3:** Comparison of mean cell area per disc (μm²) between two groups at different time intervals using independent Student's t-test SD: standard deviation

Time	Groups	N	Mean	SD	t-statistic value
24 hours	Test	8	2145	125.81	18.326
	Control	8	1221.25	67.07	
72 hours	Test	8	3756.25	177.28	18.284
	Control	8	2340	128.73	

As presented in Table 4, within the combination coating group, the mean absorbance value increased steadily across all intervals. The rise from Day one to Day three, and further to Day seven, yielded an overall statistically significant difference across time (p < 0.001). Pairwise comparisons revealed that the increases between all intervals (Day one to three, Day one to seven, and Day three to seven) were highly significant (p < 0.001), indicating progressive cell viability and proliferation. The uncoated control group also showed a gradual, statistically significant rise in mean absorbance over time (p < 0.001). However, while both groups showed time-dependent increases, the magnitude of growth was substantially higher in the combination coating group at every interval.

**Table 4:** Comparison of mean absorbance values (OD at 570 nm) for MTT assay between different time intervals in each group using repeated measures of ANOVA test followed by Bonferroni's post hoc test ^*^Statistically significant. ^a^Repeated measures ANOVA Test. ^b^Bonferroni’s post hoc test OD: optical density; ANOVA: analysis of variance; SD: standard deviation

Groups	Time	N	Mean	SD	P-value^a^	Post-hoc comparison	P-value^b^	f-value	Degrees of freedom
Test	Day 1	8	0.435	0.016	< 0.001^*^	D1 vs. D3	< 0.001^*^	1796.64	F(2, 21) = 1796.64
	Day 3	8	0.764	0.027		D1 vs. D7	< 0.001^*^		
	Day 7	8	1.173	0.029		D3 vs. D7	< 0.001^*^		
Control	Day 1	8	0.359	0.012	< 0.001^*^	D1 vs. D3	< 0.001^*^	1525.457	F(2, 21) = 1525.46
	Day 3	8	0.589	0.02		D1 vs. D7	< 0.001^*^		
	Day 7	8	0.891	0.024		D3 vs. D7	< 0.001^*^		

In Table 5, paired sample analysis of ALP activity further validated these trends. In the combination coating group, the mean ALP activity increased from 497.50 ± 22.52 U/L on Day one to 818.75 ± 29.00 U/L on Day seven, a statistically significant increment (p < 0.001) demonstrating marked osteogenic differentiation. The control group also exhibited an increase (335.00 ± 16.04 U/L to 586.25 ± 16.85 U/L; p < 0.001), though the absolute values and the magnitude of the change were lower compared to the coated group.

**Table 5:** Comparison of mean ALP activity (U/L) between Day one and Day seven in each group using paired sample t-test ^*^Statistically significant Mean difference = mean value at test - mean value at control ALP: alkaline phosphatase; SD: standard deviation

Groups	Time	N	Mean	SD	Mean Diff.	P-value	t-statistic value
Test	Day 1	8	497.5	22.52	-321.25	< 0.001^*^	24.746
	Day 7	8	818.75	29.001			
Control	Day 1	8	335	16.036	-251.25	< 0.001^*^	30.551
	Day 7	8	586.25	16.85			

Finally, as shown in Table 6, time-dependent cell spreading increased significantly within both groups. The combination coating group saw cell area expand from 2145.00 ± 125.81 μm² to 3756.25 ± 177.28 μm² between 24 and 72 hours (p < 0.001). The control group also expanded from 1221.25 ± 67.07 μm² to 2340.00 ± 128.73 μm² (p < 0.001). Ultimately, the LF and magnesium phosphate hydrogel combination coating consistently exhibited larger mean cell areas and greater expansion increments, confirming highly effective in vitro support for cell structural adhesion.

**Table 6:** Comparison of mean cell area per disc (μm²) between 24 and 72 hours in each group using paired sample t-test ^*^Statistically significant Mean difference = mean value at test - mean value at control SD: standard deviation

Groups	Time	N	Mean	SD	Mean Diff.	P-value	t-statistic value
Test	Day 1	8	2145	125.81	-1611.25	< 0.001^*^	20.964
	Day 7	8	3756.25	177.28			
Control	Day 1	8	1221.25	67.07	-1118.75	< 0.001^*^	21.800
	Day 7	8	2340	128.73			

## Discussion

The results of this in vitro study demonstrate that the application of an LF and magnesium phosphate hydrogel combination coating significantly enhances the biological response of MG-63 osteoblast-like cells on grade 4 titanium surfaces. The primary goal of surface modification in implantology is to bypass the bioinert nature of titanium and establish an interface that promotes rapid cellular integration [8]. Our findings confirm that this dual-agent coating achieves these objectives through superior cell viability, enhanced initial adhesion, and accelerated early osteogenic differentiation.

Enhanced cell viability and proliferation

The significant increase in metabolic activity observed in the experimental group, reaching a relative cell viability of 326.8% ± 8.4% by Day seven compared to 248.3% ± 7.0% in the control group (p < 0.001), underscores the potent mitogenic effect of the coating. This robust expansion to approximately 67,000 cells per disc is largely attributable to the anabolic properties of LF [13]. LF is known to stimulate the survival and proliferation of osteoblasts by activating key signaling pathways, such as the extracellular signal-regulated kinase pathway, and increasing the expression of growth factors like TGF-β and bone morphogenetic proteins [9,13]. Furthermore, the inclusion of magnesium phosphate supports this proliferative phase, as Mg ions released from the coating have been shown to increase osteoblast proliferation and provide a stable, biocompatible environment similar to human cortical bone [14].

Morphological adaptation and initial adhesion

A critical finding in our study was the 1.76-fold increase in cell spreading area within the first 24 hours. Initial cell attachment and spreading are prerequisites for long-term osseointegration, as they dictate the subsequent differentiation and mineralization phases [4]. The presence of magnesium in the coating likely facilitates this through improved protein adsorption [15]. Research indicates that magnesium-enriched titanium surfaces enhance the initial adsorption of adhesive proteins from the culture medium, which in turn promotes faster cytoskeletal reorganization and greater spreading of osteoblast-like cells [4]. This rapid morphological adaptation suggests that the hydrogel successfully modifies the titanium topography into a more favorable niche for cellular anchoring [16].

Osteogenic differentiation and clinical implications

The marked elevation in ALP activity (819 ± 28 U/L by Day seven) is a definitive indicator of early osteoblastic differentiation [17]. ALP is a key enzyme marker for the secretory phase of bone matrix formation; its upregulation in the experimental group suggests that the LF-MgP coating not only supports cell viability but also accelerates the transition of cells into a functional, bone-forming phenotype [17]. The synergistic potential of combining the antimicrobial and anabolic effects of LF with the mineral-inducing properties of magnesium phosphate is clinically significant [7]. By improving initial cell attachment and promoting faster functional activity, this modification could potentially reduce the "healing window" required before an implant can be loaded, thereby improving success rates in patients with compromised bone density [7]. Moreover, the high cell viability results confirm the excellent cytocompatibility profile of the hydrogel, suggesting its safety for future clinical applications [7].

As an initial proof-of-concept study, the experimental design was intentionally tailored to evaluate the primary biological contrast between the functionalized and conventional surfaces. Accordingly, the sample size calculation (n = 8) utilized a large anticipated effect size (Cohen’s d = 1.60), which appropriately reflects the substantial differences expected between a dual-loaded bioactive coating and plain, uncoated titanium. This approach successfully yielded robust statistical power for our core objectives. As this research progresses to evaluate narrower, single-component control arms (such as bare hydrogel, magnesium phosphate alone, or lactoferrin alone), future models will naturally incorporate adjusted effect sizes to capture more subtle comparative differences. Additionally, the current methodology prioritized high intra-assay precision by utilizing technical replicates within a strictly controlled cell culture run. Having established this strong, consistent baseline, subsequent phases of research will build upon these encouraging outcomes by incorporating independent biological repeats across multiple cell passages to confirm broad reproducibility

Regarding the experimental model, the study is strictly an in vitro laboratory simulation. We evaluated the biological response exclusively using the MG-63 human osteosarcoma-derived osteoblast-like cell line. While highly effective for initial screening, transformed cell lines inherently possess higher proliferation rates than primary human osteoblasts. Therefore, caution must be exercised when extrapolating these in vitro results to in vivo clinical osseointegration. This static model lacks the ability to mimic multi-lineage cellular interactions, localized blood coagulation, or the complex, immune-mediated "foreign body reaction" that dictates osseointegration in a living host.

While the current study establishes a robust biological proof-of-concept for this novel combination coating, future investigations will naturally expand upon these foundational findings. Having confirmed the highly favorable cellular responses, subsequent studies will seek to correlate these biological outcomes with detailed physicochemical characterizations, including micro-topographical analysis (SEM) and chemical profiling (FTIR/XPS). Furthermore, while the dip-coating technique yielded highly encouraging in vitro results, transitioning this biofunctional interface to clinical application will eventually involve evaluating the coating's mechanical bonding strength and long-term degradation kinetics under dynamic functional loading, such as simulated masticatory forces. These ongoing physical optimizations will build directly upon the excellent cytocompatibility and early osteogenic potential demonstrated here, further advancing the material toward in vivo application.

## Conclusions

The application of a lactoferrin and magnesium phosphate combination hydrogel coating significantly enhanced the in vitro biological response of human osteosarcoma-derived MG-63 cells on grade 4 titanium substrates. Compared to uncoated titanium, the modified discs demonstrated superior cytocompatibility, accelerated initial cell spreading, and marked early osteogenic differentiation. These findings validate the synergistic potential of this dual-agent surface modification in promoting a highly favorable niche for early cellular anchoring and functional maturation. While these results suggest a promising biofunctional approach for dental implant engineering, further comprehensive studies incorporating detailed physicochemical characterization, dynamic biomechanical loading, and in vivo animal models are required to fully validate its clinical efficacy and potential to support true osseointegration.
